# The Amyloid Precursor Protein Is a Conserved Receptor for Slit to Mediate Axon Guidance

**DOI:** 10.1523/ENEURO.0185-17.2017

**Published:** 2017-06-15

**Authors:** Baiping Wang, Hongmei Li, Sena A. Mutlu, Devon A. Bowser, Michael J. Moore, Meng C. Wang, Hui Zheng

**Affiliations:** 1 Huffington Center on Aging and Department of Molecular and Human Genetics, Baylor College of Medicine, Houston, TX 77030; 2 Program in Developmental Biology, Baylor College of Medicine, Houston, TX 77030; 3 Interdisciplinary Bioinnovation PhD Program, Tulane University, New Orleans, LA 70118; 4 Department of Biomedical Engineering, Tulane University, New Orleans, LA 70118

**Keywords:** amyloid precursor protein, axon guidance, receptor, Slit, corpus callosum, mice, *Caenorhabditis* elegans, explant culture, Alzheimer’s disease

## Abstract

The amyloid precursor protein (APP) is a receptor-like membrane protein. Although APP processing and β-amyloid production play a central role in Alzheimer’s disease (AD) pathogenesis, the physiological function of APP remains elusive. Here, we identify APP as a novel receptor for Slit that mediates axon guidance and neural circuit formation. APP deficiency abolishes the Slit repulsive effect in a 3D olfactory explant culture, consistent with its callosal projection deficit in vivo and reminiscent of Slit loss. Inactivation of APP ortholog APL-1 in *Caenorhabditis* elegans results in pioneer axon mistargeting and genetic analysis places APL-1 in the SLT-1 (Slit)/SAX-3 (Robo) repulsive pathway. Slit binds to APP through the E1 domain, which triggers APP ectodomain shedding and recruitment of the intracellular FE65 and Pak1 complex and associated Rac1 GTPase activation. Our study establishes APP as a novel receptor for Slit ligand mediating axon guidance and neural circuit formation.

## Significance Statement

Genetic and biochemical evidence establishes a central role of amyloid precursor protein (APP) in Alzheimer’s disease (AD). Despite extensive studies, the function of APP is still elusive. Here, we identify APP as a receptor for Slit that mediates axon guidance and neural circuit formation. We also delineate a biochemical pathway whereby Slit binding triggers APP ectodomain shedding and recruitment of an intracellular signaling complex containing FE65 and Pak1. Thus we uncover a novel function of APP in axon pathfinding; the impairment of which may contribute to neuronal dysfunction and AD.

## Introduction

Efficient neuronal communications through precise establishment of neuronal circuits are crucial for proper brain function. Abnormal connections as well as altered plasticity are associated with a variety of neurologic and neurodegenerative disorders including Alzheimer’s disease (AD; Van Battum et al., 2015). Although abundant evidence has implicated the accumulation of β-amyloid peptides (Aβ) in this process, it is worth noting that Aβ is generated through activity-dependent processing of the amyloid precursor protein (APP; Haass et al., 2012), highlighting the need for understanding AD pathogenesis in the context of APP physiologic function, particularly the establishment and maintenance of neuronal circuits and synaptic connectivity.

APP belongs to an evolutionarily conserved family of receptor-like Type I transmembrane glycoproteins consisting of APP, amyloid precursor-like proteins 1 and 2 (APLP1 and APLP2) in mammalian system (Kang et al., 1987). Numerous studies have implicated the role of APP in the regulation of neurite outgrowth (Perez et al., 1997; Young-Pearse et al., 2008; Rama et al., 2012), migration of neuronal precursor cells (Herms et al., 2004; Young-Pearse et al., 2007), cell adhesion (Soba et al., 2005), synaptogenesis (Wang et al., 2005; Priller et al., 2006; Wang et al., 2009), and adult neurogenesis (Demars et al., 2011; Wang et al., 2014). Loss-of-function studies in mice revealed low frequency of callosum agenesis in *APP* null mice. This phenotype is only present on 129 but not C57 background, indicating the presence of strain-specific modifiers (Magara et al., 1999). Analysis of *APP* and *APLP2* double knock-out (dKO) or mice deficient in all three APP members showed axon terminal sprouting of the neuromuscular junction and over-migration of embryonic forebrain neurons (Herms et al., 2004; Wang et al., 2005). However, the genetic redundancy of the APP family of proteins and the early postnatal lethality of the *APP/APLP2* double null mice hindered further mechanistic and functional understanding of APP in adult brain. In *Caenorhabditis* elegans, APP has one single ortholog APP-like 1 (APL-1; Daigle and Li, 1993), thereby offering an attractive genetic system to investigate the function of APP/APL-1 *in vivo*. Yet, loss of APL-1 disrupts molting and results in larval lethality, and the neuronal aspects of APL-1 function, particularly in neural circuitry, has been lacking. Importantly, APP’s putative ligand, ligand triggered receptor signal transduction pathway and the relationship to the fundamental role of APP in brain circuitry remain to be established.

Significant progress has been made in understanding the pathways involved in axon guidance, with classic guidance families include Netrins, Slits, Semaphorins and Ephrins and their respective neuronal receptors, DCC, Robo, Neuropilins/Plexins and Ephs (Dickson, 2002). Among these, the Slit family proteins (Slit) and Roundabout (Robo) receptors are evolutionary conserved chemorepulsive axon guidance molecules primarily known for their function in regulating midline crossing of major forebrain cortical axonal tracts (Bagri et al., 2002; Shu et al., 2003), commissural axons in the spinal cord (Long et al., 2004) and olfactory bulb (OB) axons (Nguyen-Ba-Charvet et al., 2002). However, it is unlikely that Robo can account for all Slit functions. For instance, the commissural axon guidance phenotype in Robo1 and Robo2 deficient mice are less severe than mice lacking all Slits (Jaworski et al., 2010). Similarly in *Drosophila*, loss of Slit leads to complete collapse of all CNS axons whereas loss of Robo results in a milder axon misrouting at the midline (Kidd et al., 1999; Jaworski et al., 2010).

Using genetic studies in both *C. elegans* and mice and corroborated with biochemical and functional evidence, we reveal APP as a new receptor for Slit and identify its intracellular signaling components that mediate Slit-induced axon pathfinding.

## Materials and Methods

### Mice

The *APP* and *APLP2* germline and conditional knockout mice (Wang et al., 2009) have been described previously. All mice used for this study have been backcrossed to C57BL/6 background for at least ten generations. Both female and male mice were used. All experiments were performed in accordance with procedures approved by the Institutional Animal Care and Use Committee of Baylor College of Medicine and guidelines established by the National Institutes of Health.

### Nematode strains and RNAi in *C. elegans*

The following strains were obtained from the Caenorhabditis Genetics Center which is funded by NIH Office of Research Infrastructure Programs (P40 OD010440): IC136 *zdIs5[mec-4::gfp]* I; *vab-1(dx31)* II, TU3401 *sid-1(pk3321); uIs69[unc-119p::sid-1]*, CX3171 *sax-3(ky200)*, CX5000 *slt-1(eh15)*, MT324 *unc-40(n324)*, and NW434 *unc-6(ev400)*. The following worms were derived using standard genetic procedures: MCW451 *sid-1(pk3321); uIs69; zdIs5*, MCW430 *sax-3(ky200); sid-1(pk3321); uIs69; zdIs5*, MCW453 *slt-1(eh15); sid-1(pk3321); uIs69; zdIs5*, MCW518 *unc-40(n324); sid-1(pk3321); uIs69*; zdIs5, MCW519 *unc-6(ev400); sid-1(pk3321); uIs69; zdIs5*. Worms were raised and maintained at 20°C using standard techniques unless otherwise noted.

RNAi clones from the Ahringer library transformed into HT115 cells were streaked on Lysogeny broth (LB) plate with 50 μg/ml ampicillin and 10 μg/ml tetracycline; 5 ml of bacteria miniculture in LB with 50 μg/ml carbenicillin were grown at 37°C with agitation for 14 h. Bacteria were concentrated by centrifugation to 1 ml and then added to Nematode growth media (NGM) plates with 5 mM Isopropyl β-D-1-thiogalactopyranoside for overnight induction. Worms were synchronized by standard egg preparation procedures followed by hatching overnight in M9 buffer. L1 worms were fed with RNAi bacteria with either the control vector L4440, or plasmids carrying RNAi of gene of interest. L1 worms were grown at 20°C (except for *sax-3(ky200)* line at 25°C) on RNAi food for 48–60 h before counting for AVM axon guidance defect.

### AVM axon guidance analysis

Animals were mounted on agarose pads, anaesthetized with 0.2% sodium azide in M9 buffer, and examined under 20x objective lens on a Leica TCS confocal microscope. Axons of AVM neurons were visible with the transgenic array *zdIs5[Pmec-4::gfp]*. Worms were counted as mutant if the AVM axon projected in the anterior or the posterior direction erroneously, for at least ∼15 μm (more than three AVM cell body lengths) before projecting to the ventral side. AVM mutant percentage reported are average of three to six independent set of experiments, with a minimum of 200 worms. The experimenters were blinded of genotypes.

### Immunofluorescence staining

Brains were harvested, postfixed in 4% paraformaldehyde, dehydrated with 30% sucrose in PBS, and serially sectioned at 40 μm on a vibratome (Leica). For immunofluorescence, sections were permeabilized in 0.1% Triton X-100/PBS for 10 min and blocked with 4% normal goat serum in PBS/0.1% Triton X-100 for 1 h at room temperature. Sections were then incubated with primary antibodies in 2% serum in PBS/0.1% Triton X-100 overnight at 4°C. The primary antibodies used in this study were rabbit anti-glial fibrillary acidic protein (GFAP; G9269, Sigma); chicken anti-neurofilament H (AB5539, Millipore Bioscience Research Reagents); rat anti-L1 (MAB5272, Millipore Bioscience Research Reagents); rat anti-Ctip2 (ab18465, AbCam), rabbit anti-APLP2 (gift from G. Thinakaran), APP Y188 (AbCam) and 6E10 (Covance). Sections were then washed and incubated with Alexa Fluor 488- or Alexa Fluor 596-conjugated secondary antibody (Invitrogen) for 1 h at room temperature. For myelin staining, mouse coronal sections were incubated with FluoroMyelin Green fluorescent myelin stain (1:300, Invitrogen) for 20 min at room temperature. After washing with PBS, sections were mounted with Vectashield Mounting Medium with DAPI (Vector Laboratories). Images were captured using a Leica confocal microscope.

### Plasmid constructs, cell culture, and cell surface binding assay

Full-length (FL) human APP_695_, APPΔE1, APPΔE2, and APPΔC expression constructs were described previously (Wang et al., 2009). Human Slit2 tagged with c-Myc (Slit2-Myc) and rat Robo1 tagged with the hemagglutinin (Robo1-HA) were described previously (Li et al., 1999). Constructs containing Slit2 N-terminal (Slit2N) and C-terminal (Slit2C), according to Nguyen Ba-Charvet et al. (2001), were generated using the Q5 Site-Directed Mutagenesis kit (New England Biolabs) and confirmed with sequencing. FL Slit2 fused N terminally to the human alkaline phosphatase sequence was generated from Ntn5.a-AP-His (Addgene) after excision of Netrin 5 from the vector. Other constructs used in this study include HA-Fe65 (Cao and Sudhof, 2001) and Myc-Pak1 (Duman et al., 2013). pRK5-Myc-Rac1 were purchased from Addgene. The ADAM10 inhibitor GI254023X (Sigma) was described previously (Hundhausen et al., 2003).

HEK293 cells were cultured in DMEM supplemented with 10% fetal bovine serum. For cell surface binding assay, HEK293 cells plated on glass coverslips were transiently transfected with either APP_695_, Robo1-HA or APP deletion constructs using the lipofectamine reagent (Invitrogen). Twenty-four hours later, the cells were incubated with serial dilutions of previously dosed supernatants from HEK293 cells expressing Slit2-Myc supplemented with 1 μg/ml heparin for 1.5 h at 37°C. After washing three times in PBS, cells were fixed in 4% PFA for 20 min at room temperature and permeabilized with 0.1% NP40 in PBS for 15 min. Double immunofluorescent labeling of transfected receptors and bound Slit2 were conducted using the anti-APP antibody (4G8, Biolegend), mouse HA antibody (6E2, Cell Signaling) and rabbit Myc antibody (A14, Santa Cruz Biotechnology) followed by incubation with secondary antibodies. For colocalization quantification, images were background-subtracted and region of interest of one cell was delineated. Quantitative colocalization analysis evaluating the average proportion of Slit2 signals colocalized with Robo, FL, or mutant APP was performed over the entire fluorescence images using the plugin ‘Coloc 2’ on the Fiji software (ImageJ). The thresholded Manders M1 coefficient was expressed and compared between groups.

For quantitative binding assays, HEK293 cells were transfected with either FL APP or HA-Robo. Twenty-four hours later, the cells were incubated with serial dilutions of previously dosed supernatants from HEK293T cells expressing AP-tagged Slit2 and 1 μg/ml heparin for 2 h at 37°C. After washing with cold PBS, Alkaline Phosphatase activities in the cell lysates were measured with the Alkaline Phosphatase Assay kit (AbCam). Optical density at 405 nm was determined using a SpectraMax i3x microplate reader (Molecular Devices). Binding curves were fitted using the Hill equation, and corresponding dissociation constant (*K*_d_) values were calculated with Prism 6 software (GraphPad).

Cortical neurons from postnatal day 0 (P0) mouse were plated in six-well poly-D-lysine coated plates at 10^6^ cells/well for 7 days *in vitro* (DIV). In the coculture assay, the HEK293 cell expressing Slit2 were seeded on cell culture inserts (Costar) and transferred to 6 DIV primary neurons. The conditioned media (CMs) and cell lysates were collected for Western blot analysis 24 h later.

### Western blotting and immunoprecipitation (IP)

Co-IP experiments were performed as described previously (Wang et al., 2007). Western blot analysis was performed using the following primary antibodies: anti-APP [APPc (Wang et al., 2009) and 22C11, Millipore], mouse HA antibody (6E2, Cell Signaling) and rabbit Myc antibody (A14, Santa Cruz Biotechnology), anti-Slit2N (GeneTex), anti-Rac1 (BD Transduction Laboratories).

For glutathione S-transferase (GST) pull-down, the GST-APPE1 fusion construct containing amino acids 22–189 of APP extracellular E1 domain was generated in pGEX-4T-1 (GE Life Science). The fusion proteins were expressed in bacteria and purified by glutathione-agarose as described (Thermo Fisher). GST binding assays were done as previously described (Borg et al., 1996). Purified GST, GST-APPE1 bound to glutathione agarose beads were incubated with CM from HEK293 cells overexpressing Myc-tagged Slit2 for 3 h at 4°C. The beads were washed three times and bound proteins were eluted with 50 μl of elution buffer (50 mM glutathione and 50 mM Tris-Cl, pH 8.0) and then analyzed by SDS–PAGE followed by Western blotting.

For APP processing assays, HEK293 cells plated in six-well plates were transiently transfected with Slit2-Myc or empty vector together with APP_695_ or APP deletion constructs. Twenty-four hours after transfection, media (DMEM + 10% FBS) were replaced. At 48 h after transfection, media were collected and centrifuged for analysis of soluble APP (APPs). Western blot analysis was performed using antibodies against total sAPP (22C11), human or rodent α-secretase cleavage-specific sAPP (sAPPα, 2B3 and 597, respectively, Immuno-Biological Laboratories). Cells were lysed for measurement of holoAPP and APP-C-terminal fragment (CTF) using the APPc antibody.

### Biomimetic 3D OB explant culture and axon guidance assay

Dual hydrogel micropatterned constructs, designed to present a bipolar choice point that allows neurite outgrowth to respond to different soluble cues, was created using digital projection photolithography using a digital micromirror device (Texas Instruments) and methods similar to those previously described (Horn-Ranney et al., 2013). Briefly, a hydrogel precursor solution consisting of 10% (w/v) polyethylene glycol dimethacrylate (PEGDA, MW 1000, Polysciences) in PBS with 0.5% (w/v) Irgacure 2959 (Ciba Specialty Chemicals) as the photoinitiator was prepared. A total of 500 µl of the precursor solution was added to a Transwell Permeable insert (Corning). The hydrogel precursor solution was exposed to 40 s of ultraviolet light (320-500 nm) patterned by a photomask to initiate crosslinking throughout the depth of the solution thereby creating an outer 3D growth restrictive hydrogel.

A commercially available hydrogel kit, Hystem-HP (ESI-BIO), was used as the inner growth permissive hydrogel. Hystem-HP utilizes thiolated hyaluronan, heparin, and collagen in combination with a thiol-reactive PEGDA to undergo *in situ* gelling. The Hystem-HP components were prepared according to the manufacturer’s instructions and then mixed in a 2:2:1 ratio of Heprasil:Gelin-S:Extralink. The mixed Hystem-HP solution was carefully added to the center bipolar void within the micropatterned PEG hydrogels using a micropipette. The solution was allowed to gel in a sterile environment at room temperature for 30 min. The two circular voids, one on each end of the bipolar channel, were left as empty wells (i.e., no Hystem-HP was added) to allow for the addition of cells during experiments. Completed inserts were washed overnight in a 2% (v/v) anti/anti solution in PBS to allow rehydration and disinfection of the constructs.

OBs were dissected from embryonic day 15 (E15) embryos, cut into small pieces (∼200 µm in diameter), and carefully placed directly on top of the Hystem-HP and then gently pressed into the Hystem-HP gels within the circular region. Immediately after the addition of OB explants, HEK293 cells expressing Slit2-Myc or empty vector were introduced to the construct by adding the cells to the empty wells at the end of opposite channels. Explants were cultured in Neurobasal medium with 10% B27 supplement, 1% penicillin/streptomycin, 0.5 mM L-glutamine at 37°C, and 5% CO_2_ for 72 h.

Explants were fixed in 4% paraformaldehyde for 2 h. Neurite staining was performed using neuron-specific β III tubulin primary antibody (AbCam). Primary and secondary staining was conducted in PBS with 0.1% Triton X-100 and 2% BSA for 48 h. Images were captured using a Leica confocal microscope. ImageJ (National Institutes of Health) was used for postprocessing of images. Maximum intensity Z-stacks were obtained from the confocal images. To quantify neurite growth, automatic thresholding (mean) was used, followed by pixel volume analysis. A guidance ratio as previously described (Curley et al., 2014) was used to determine the effect of Slit2 on neurite growth.

### Rac1 activation assay

HEK293 cells were transfected with APP, starved for 2 h in OptiMEM medium and incubated with supernatants from HEK293T cells transfected with Slit2 construct. Rac1 activation was measured using the Rac1 activation assay kit (Millipore). Briefly, cell lysates were incubated with GST fusion protein, corresponding to the p21-binding domain (PBD, residues 77-151) of human Pak1 bound to glutathione agarose (Millipore) for 1 h at 4°C, allowing for coprecipitation of GTP-bound Rac1. Pellets were washed three times, and inputs and pellets were analyzed by Western blotting with the anti-Rac1 antibody.

### Statistics

Statistical significance was established by Student’s *t test* and two-way ANOVA followed by the Bonferroni multiple comparison posttest, using Excel or GraphPad Prism. All data were reported as mean ± SEM; *p < 0.05, **p < 0.01, and ***p < 0.001.

## Results

### Expression of APP and APLP2 in developing forebrain axonal tracts and projection neurons

**Figure 1.**
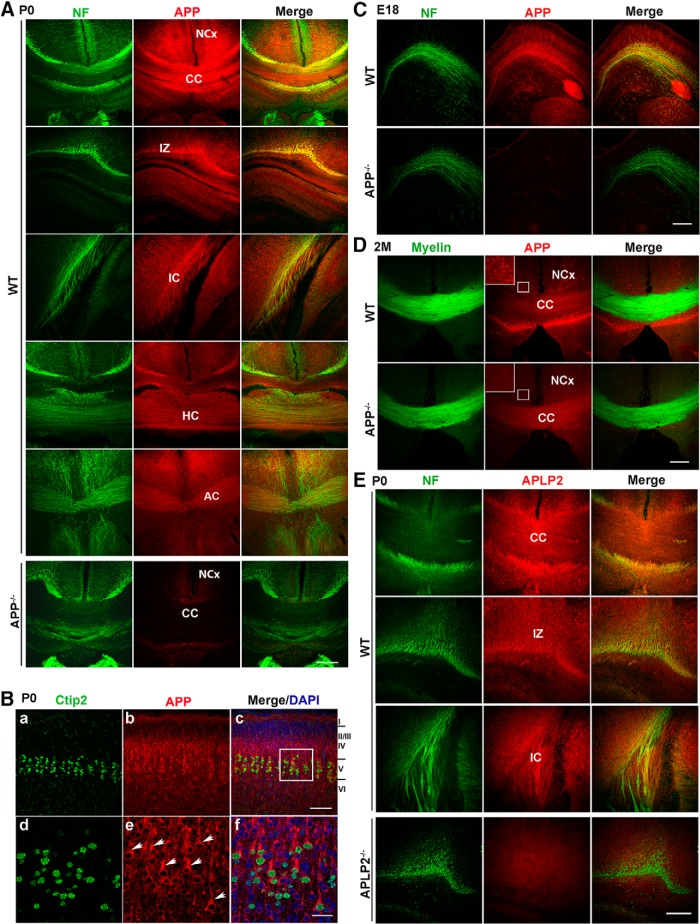
Expression of APP in developing axons and projection neurons. ***A***, APP and neurofilament (NF) double immunostaining in P0 WT mouse brains, revealing abundant APP expression in axonal tracks of CC, IZ, and IC, HC, and AC. APP^−/−^, a corresponding CC section of *APP* null mice; NCx, neocortex. ***B***, APP and Ctip2 double immunostaining in P0 WT mouse cortex (with Layers I-VI marked on right). ***d–f*** are high resolution images of bracketed areas in ***c*** highlighting prominent expression of APP (red, arrow heads) in Ctip2-positive Layer V projection neurons (green). ***C***, NF and APP double staining, showing positive axonal expression of APP in E18 WT, but not APP^−/−^, mouse brains. ***D***, Two-month-old WT and APP^−/−^ brain sections were double stained with APP and FluoroMyelin Green. Bracketed areas are enlarged view showing APP expression in the neuronal cell bodies. ***E***, APLP2 and NF double immunostaining in P0 WT mouse brains, revealing APLP2 expression in axonal tracks of CC, IZ, and IC. APLP2^−/−^, a corresponding IC section of *APLP2* null mice. Merge: overlay of the single-stained panels. Scale bars, 100 μm [***A***, ***B*** (***a–c***), ***C–E***] and 20 μm [***B*** (***d–f***)].

To assess whether APP plays a role in axon guidance, we first examined the expression pattern of APP in developing forebrain. Double immunofluorescence staining of P0 mouse brain sections for APP and neurofilament revealed that APP was highly expressed in corpus callosum (CC), the major axonal projection pathway joining the two hemispheres of the mammalian brain (Fig. 1*A*). Moreover, axonal tracts associated with subcortical projections in the intermediate zone (IZ), internal capsule (IC), hippocampal commissure (HC), and anterior commissure (AC) were strongly labeled with APP (Fig. 1*A*).

In addition to axonal projections, APP was also found to be highly expressed in cell bodies of pyramidal neurons (Fig. 1*B*). Colabeling of APP with Ctip2, a marker for cortical projection neurons in Layer V which contains callosal neurons that project to the contralateral hemisphere as well as neurons that extend axons to subcortical targets, revealed that APP was particularly prominent in cell bodies of Ctip2-positive neurons (Fig. 1*B*, arrowheads). Developmentally, callosal axons from Layer V first project to the contralateral targets, initiated at approximately E16, with callosal axons from the upper layers following the path (Ozaki and Wahlsten, 1998). Examination of APP at embryonic stage 18 (E18) showed early axonal APP staining during the formation and crossing of axons (Fig. 1*C*). High levels of APP expression in projection neurons persisted through adulthood (Fig. 1*D*, bracketed areas). In contrast, the expression of APP in axonal tracks were significantly downregulated in adult mouse brains (Fig. 1*D*, CC and compare with Fig. 1*A*, CC), indicating a tight regulation of APP expression in axons during development. Costaining of P0 brains with anti-neurofilament and anti-APLP2 antibodies detected APLP2 immunoreactivity along the axonal tracks including callosal axons similar to that of APP (Fig. 1*E*). The temporal and spatial patterns of APP and APLP2 expression indicate that the APP family of proteins may play important but redundant roles in the pathfinding of forebrain axonal tracts and cortical circuit formation.

### Severe cortical callosal projection deficits in APP/APLP2 mutant mice

**Figure 2.**
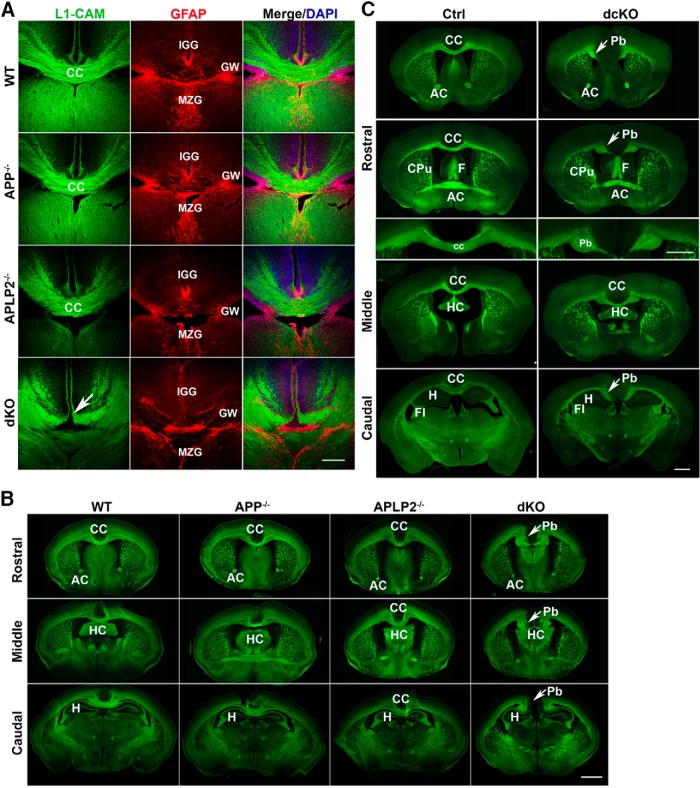
Abnormal development of the CC in APP/APLP2 double mutant mice. ***A***, Presence of midline glial structures but severe cortical callosal projection deficits in *APP/APLP2* dKO mice. Coronal brain sections of P0 littermates of WT, APP^−/−^, APLP2^−/−^, and APP/APLP2 dKO mice were colabeled with L1-CAM and GFAP, the latter identifies all three midline glial populations. GW: glial wedge; IGG: indusium griseum glia; MZG, midline zipper glia. Arrow indicates failed midline crossing of CC in dKO mice. ***B***, Neurofilament staining of P0 rostral, middle, and caudal coronal sections of WT, APP^−/−^, APLP2^−/−^, and dKO mice, showing similar CC structures in WT, APP^−/−^, and APLP2^−/−^ mice but failed midline crossing and formation of Pbs in dKO mice. ***C***, FluoroMyelin Green staining of serial rostral to caudal sections of adult mouse brains of control (Ctrl) and *APP/APLP2* double conditional (dcKO) mice demonstrating dysgenesis of CC and appearance of ectopic Pb (arrows) in dcKO mice. CC: corpus callosum; AC: anterior commissure; HC: hippocampal commissure; CPu, caudate putamen; F, fornix; Fi, fimbria; H, hippocampus. Scale bars, 100 μm (***A***) and 1 mm (***B***, ***C***).

We thus sought to examine whether axon guidance of the major forebrain tracks were impaired in mice deficient in *APP*, *APLP2*, or both combined. L1-CAM staining of P0 mouse brains revealed that, consistent with the overlapping expression patterns and compensatory activities, the *APP* and *APLP2* single knockout brains were overtly normal (Fig. 2; APP^−/−^ and APLP2^−/−^). However, in *APP/APLP2* dKO mice, the axons in the CC failed to cross the midline (Fig. 2*A*, dKO). GFAP labeling of the same brain sections for midline glial structures that provides substrates for callosal axons to traverse showed the presence of all three midline glial populations, namely, the glial wedge (GW), the indusium griseum glia (IGG), and the midline zipper glia (MZG) in dKO brains. We noted that the IGG were displaced more ventrally relative to the GW in the dKO brains (Fig. 2*A*, dKO). Given the minimum expression of APP/APLP2 in these glial structures, it is likely that the mispositioning is secondary to the formation of the large axonal fascicles and the lack of a definitive CC as observed in *Robo1* null mice (Andrews et al., 2006).

Further examination of P0 CC structures in serial neurofilament-stained coronal sections of P0 wild-type (WT), APP^−/−^, APLP2^−/−^, and *APP/APLP2* dKO mice from rostral to caudal directions showed that, in WT, APP^−/−^, and APLP2^−/−^ mice, the CC was clearly connected throughout the cerebral hemispheres (Fig. 2*B*; CC in WT, APP^−/−^, and APLP2^−/−^). In dKO mice, however, the medial projection axons failed to cross the midline and instead turn vertical to form axon fascicles known as Probst bundles (Pbs; Fig. 2*B*, Pb in dKO). AC and HC pathways were not overtly affected.

The development of CC continues postnatally. Since the germline *APP/APLP2* dKO mice are early postnatal lethal, to ascertain whether the CC phenotype detected at P0 persists to adulthood, we chose to analyze the *APP* and *APLP2* double conditional knock-out mice (*APP* flox/flox; Nestin-Cre; APLP2^−/−^, or dcKO), which are viable despite lacking APP and APLP2 in the brain (Wang et al., 2009). Staining of 2-month-old control and dcKO brain sections with FluoroMyelin Green to mark myelinated axons revealed similar failed midline crossing of CC and malformation of Pbs in 61.5% of the mutants (16/26; Fig. 2*C*).

The presence of CC phenotype in both germline and conditional *APP/APLP2* double mutant mice demonstrate that the APP family of proteins cooperate to control proper formation of prominent axon tracts in the mammalian forebrain. Similar defects have been observed in several targeted mutations of genes directly involved in midline axon guidance particularly Slit/Robo (Lopez-Bendito et al., 2007; Unni et al., 2012) and Netrin/DCC (Serafini et al., 1996; Fazeli et al., 1997), prompting us to hypothesize that APP may genetically and/or biochemically participate in these pathways.

### APL-1 mediates pioneer axon guidance through SLT-1/SAX-3 signaling independent of UNC-6/UNC-40

**Figure 3.**
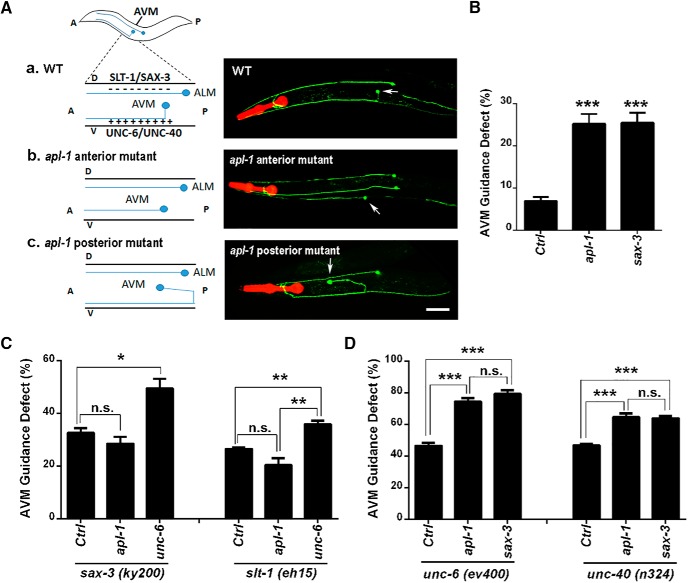
APL-1 deficiency in *C. elegans* affects AVM neuron ventral axon guidance. ***A***, Schematic diagram and representative fluorescence images of mechanosensory ALM and AVM neurons (arrows) visualized by *zdIs5[mec-4:gfp]* in WT (***a***) and *apl-1* knock-down worms (***b***, ***c***). In WT *C. elegans*, dorsally expressed SLT-1/SAX-3 repels whereas ventrally expressed UNC-6/UNC-40 attracts AVM axons to grow toward ventral nerve cord before extending anteriorly to the nerve ring. In worms with *apl-1* RNAi, AVM axons extend anteriorly (***b***) or posteriorly (***c***) instead of growing ventrally first. A, anterior; P, posterior; D, dorsal; V, ventral. Scale bar, 50 μm. ***B***, Quantification of AVM axon guidance deficits in worms (strain MCW451) treated with L4440 empty vector control (Ctrl), *apl-1*, or sax-3 RNAi for 48 h starting at L1 stage. ***C***, Quantitation of the AVM axon guidance defects in *sax-3(ky200)* or *slt-1(eh15)* mutant worms treated with control, *apl-1* or *unc-6* RNAi. *apl-1* RNAi cannot further increase AVM guidance defects in *sax-3(ky200)* or *slt-1(eh15)* mutant worms. ***D***, Quantitation of the AVM axon guidance deficits in *unc-6(ev400)* or *unc-40(n324)* mutants treated with control (Ctrl), *apl-1*, or *sax-3* RNAi for 48 h. Data represent average ± SEM. Each experiment was repeated in five to six independent batches with GFP RNAi showing invisible AVM neurons in >85% of the worms in each batch. Total number of worms counted for each genotype was between 197 and 643. One-way ANOVA (***B***) and two-way ANOVA followed by the Bonferroni multiple comparison posttest (***C***, ***D***) were used. *p < 0.05; **p < 0.01; ***p < 0.001; n.s., nonsignificant.

To test a possible genetic interaction of APP in Slit/Robo- or Netrin/DCC-mediated axon guidance pathways, we turned to the nematode *C. elegans* as these signaling pathways are well-conserved between *C. elegans* and higher organisms. The mechanosensory AVM neurons in *C. elegans* are ideal for studying ventral guidance of axon projections as their axons are guided by both repulsive and attractive signaling pathways acting in parallel. Specifically SLT-1 (Slit)/SAX-3 (Robo) directs repulsive guidance from the dorsal body wall (Hao et al., 2001), whereas UNC-6 (Netrin)/UNC-40 (DCC) mediates attractive guidance toward the ventral side (Yu and Bargmann, 2001). In WT worms of first larval stage (L1), the AVM axons are initially attracted ventrally to the ventral nerve cord. Once they reach the body wall, the axons turn anteriorly and extend to the nerve ring (Fig. 3*A*, WT). Disruption of these guidance pathways cause AVM to project erroneously to anterior or posterior directions from the cell body instead of extending ventrally first.

To test whether APL-1 deficiency in *C. elegans* leads to deficits in pioneer axon pathfinding of AVM neurons, we took an RNAi approach since the AVM neurons develop postembryonically (Goodman, 2006), and the *apl-1* null mutants are L1 lethal (Hornsten et al., 2007; Wiese et al., 2010). We crossed transgenic worms expressing GFP under the *mec-4* promoter to mark mechanosensory receptors including the AVM to TU3401 strain to render the neurons sensitive to RNAi. We found that knocking-down *apl-1* in synchronized L1 worms resulted in viable adults and ∼30% of AVMs displayed ventral guidance defects, comparable to that of *sax-3* RNAi (Fig. 3*A*,*B*). GFP RNAi was conducted simultaneously in all experiments to control the RNAi efficiency. Only data from batches with no appreciable AVM fluorescence by GFP RNAi were counted. As observed by others, WT worms under control RNAi showed minor perturbations in AVM axon projection (Bernadskaya et al., 2012), but the degree was significantly lower than *apl*-1 or *sax-3* RNAi (Fig. 3*B*).

We next performed genetic analysis by examining the AVM ventral guidance in double mutants defective in either SLT-1/SAX-3 repulsive guidance or UNC-6/UNC40 attractive guidance, together with APL-1 deficiency. We first tested whether APL-1 functions in the SLT-1/SAX-3 repulsive signaling pathway by feeding the *sax-3(ky200)* or *slt-1(eh15)* mutant worms with control or *apl-1* RNAi and assessing the ventral guidance of AVM neurons. We found that on control RNAi, 33% of *sax-3(ky200)* loss-of-function mutants displayed AVM projection defects at the restrictive temperature 25°C (Fig. 3*C*). *apl-1* knockdown failed to further enhance these deficits (Fig. 3*C*). As a positive control, *unc-6* RNAi in *sax-3(ky200)* mutants resulted in additional increase in AVM guidance defect (Fig. 3*C*). Similar results were obtained when the same set of RNAi were applied in *slt-1*(*eh15*) loss-of-function mutants (Fig. 3*C*).

To further examine the involvement of *apl-1* in the AVM guidance pathway, we applied *apl-1* RNAi in *unc-6(ev400)* or *unc-40(n324)* null mutant worms, in which the attractive axon guidance signaling pathway was abolished. We found that, compared with control RNAi, *apl-1* RNAi in both *unc-6(ev400)* and *unc-40(n324)* background significantly enhanced the AVM guidance deficits to the degree similar with *sax-3* RNAi (Fig. 3*D*). The data combined establish that *apl-1* and *slt-1/sax-3* act in the same repulsive pathway in parallel to the *unc-6/unc-40* attractive guidance.

### APP mediates axon repulsive function of Slit2 in a 3D olfactory explant culture

**Figure 4.**
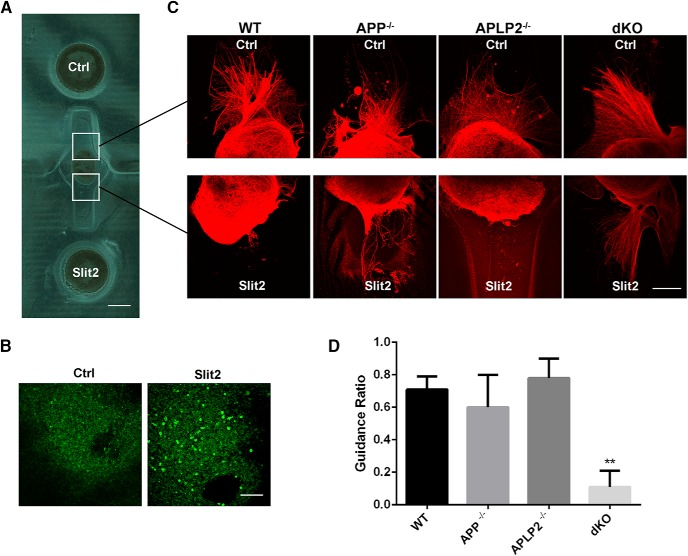
APP mediates Slit repulsive signal in an OB explant axon guidance assay. ***A***, Micropatterned dual hydrogel device for OB explants and HEK293 cell coculture. OB explant was placed in the middle of the chamber. Untransfected HEK293 cells (Ctrl) and cells transfected with Slit2-Myc (Slit2) were placed in the wells at each end of the neurite growth chamber. ***B***, Slit2 expression detected by staining with the Myc antibody. ***C***, Anti-β-tubulin staining of E15 OB explants from WT, APP, and APLP2 single (APP^−/−^; APLP2^−/−^) or dKO mice cocultured with Ctrl or Slit2-expressing cells for 72 h. Axons from WT, APP^−/−^, APLP2^−/−^ OB explants grew away from Slit2-expressing cells. In contrast, axons from dKO OB explants grew symmetrically. ***D***, Quantification of APP/APLP2 effect on Slit2-dependent axon guidance ratio. Statistical comparison was done by one-way ANOVA. For each genotype, a total of 4-10 OB explants were evaluated. Scale bars, 500 μm (***A***) and 100 μm (***B***, ***C***).

To directly test whether APP could serve as a functional receptor mediating Slit repulsive activity, we employed a 3D *ex vivo* axon guidance assay using OB explants, as previous studies have shown that Slit2 can act as a repulsion factor in the olfactory system (Li et al., 1999). We cocultured E15 OB explants from WT, *APP* null, *APLP2* null, or *APP/APLP2* dKO mice in a micropatterned dual hydrogel device with control and Slit2-expressing cell aggregates (Fig. 4*A*,*B*), which allow for exposure of neurites to the guidance cues provided by the cell aggregates in a highly quantifiable and controlled manner (Horn-Ranney et al., 2013). The device contains a micro-mold formed by photocrosslinked PEG hydrogel to serve as a restrictive border with a second Hystem-HP gel inside which can be used as a 3D supportive matrix for axon growth. The geometry of the micropatterned construct was designed to include two circular cell reservoirs for placing cell aggregates at a distance of 500 μm away from the explant to allow neurite growth. Neurites from the explant grew into the permissive Hystem-HP gel and could be visualized by βIII-tubulin staining (Fig. 4*C*) and quantified from thresholded confocal image stacks (Fig. 4*D*). When WT OB were cultured, axons were repelled from Slit2-expressing cells (Fig. 4*C*, WT, Ctrl vs Slit2, and qualified in Figure 4*D*). This was also the case when *APP* and *APLP2* single null explants were cultured (Fig. 4*C*,*D*, APP^−/−^; APLP2^−/−^). In contrast, axons from dKO OB explants extended in a radial pattern toward both control and Slit2-expressing cells in a symmetric pattern (Fig. 4*C*, dKO and qualified in Figure 4*D*). Thus, APP and APLP2 play functional but compensatory roles in Slit-mediated axon repulsion.

### Slit binds to APP through the extracellular E1 domain

**Figure 5.**
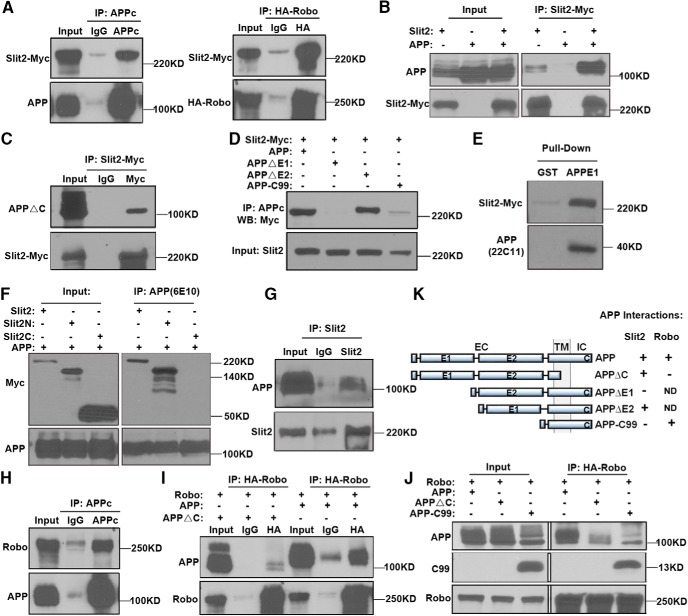
Biochemical analysis of APP, Slit, and Robo interactions. ***A***, HEK293 cells coexpressing APP and Slit2-Myc (left), or Robo-HA and Slit2-Myc (right) were immunoprecipitated with APPc or anti-HA antibodies for APP and Robo, respectively, revealing positive interaction of APP with Slit2 similar to Robo. ***B***, Reciprocal IP of Slit2-Myc- and APP-cotransfected cells (Slit2 +/APP +) using the anti-Myc antibody pulled-down APP. Single Slit2 or APP transfections (Slit2 +/APP – or Slit2 −/APP +) were used as controls. The data revealed that Slit2 can also pull-down endogenous APP although at a level significantly less than overexpressed APP. ***C***, Cotransfection of Slit2-Myc and APPΔC and IP for Slit2-Myc showed a positive interaction of Slit2 with APPΔC. ***D***, Cotransfection of Slit2-Myc with APP mutants as indicated and IP using the APPc antibody showed that Slit2-Myc is pulled-down with FL APP (APP) and APPΔE2 but not APPΔE1 and APP-C99. ***E***, *In vitro* GST pull-down using a GST-APPE1 domain fusion protein showing that Slit2-Myc can be pulled-down from CM of Slit2-Myc-transfected HEK293. Pull-down with GST backbone was used as a negative control. ***F***, Co-IP of HEK293 cells transfected with APP, Slit2, Slit2N, or Slit2C fragments revealed that FL Slit2 and Slit2N but not Slit2C coimmunoprecipitated with APP. ***G***, IP of P0 WT mouse brain lysates with an anti-Slit2 antibody and Western blotting using the anti-APP antibody 22C11 revealed the interaction of endogenous APP and Slit2. ***H***, FL APP was coexpressed with HA-tagged Robo and immunoprecipitated with APPc antibody. Robo was coimmunoprecipitated with APP. ***I***, Reciprocal IP using an anti-HA antibody to immunoprecipitate Robo also pulled-down FL APP, but not APP mutant deleting the C-terminal domain (APPΔC). ***J***, Coexpression of HA-Robo with APP C-terminal construct (APP-C99) and IP with the anti-HA antibody revealed that APP-C99 coimmunoprecipitated with HA-Robo. ***K***, Schematic diagram showing APP mutant constructs and their interactions with Slit2 or Robo. Positive and negative interactions were indicated as (+) and (-), respectively. ND, not done.

The above studies raised the possibility that APP may function as a novel receptor for Slit. We thus tested a possible interaction between APP and Slit by cotransfecting the cDNAs expressing FL human APP and Myc-tagged Slit2 to HEK293 cells followed by IP and Western blotting using anti-APP or Myc antibodies. HA-tagged Robo was included as a positive control. We found that Slit2 was pulled-down with both anti-APP and HA antibodies (Fig. 5*A*). Reciprocal IP using an anti-Myc antibody also pulled-down APP (Fig. 5*B*).

FL APP contains an extracellular domain (EC) that can be subdivided to a cysteine-rich globular domain (E1) and a region rich in acidic residues (E2), a transmembrane domain (TM) and an intracellular domain (IC). To dissect the APP sequences mediating Slit binding, we constructed APP mutants deleting the C-terminal domain (APPΔC), extracellular E1 (APPΔE1) or E2 domains (APPΔE2) and APP mutant deleting the N-terminal domain (APP-C99; Fig. 5*K*). Co-IP experiment showed that APPΔC retained interaction with Slit2, consistent with the extracellular ligand-receptor interaction prediction (Fig. 5*C*). Moreover, expression of Slit2 with APPΔE1 or APPΔE2 and IP with the anti-APP antibody (APPc) revealed that Slit2 coimmunoprecipitated with APPΔE2 whereas APPΔE1 abolished the interaction (Fig. 5*D*). Thus, APP binds to Slit2 through its E1 domain. Next, we conducted *in vitro* GST pull-down assays to test for an interaction between the E1 domain of APP and Slit2 by incubating the GST-APPE1 fusion protein (APPE1) with CM from HEK293 cells overexpressing Myc-tagged Slit2. GST pull-down followed by Western blot analysis using the anti-Myc antibody showed that only the GST fused with APPE1, but not to GST alone, was able to pull-down Slit2 (Fig. 5*E*). To delineate whether APP interacts with Slit2 through its N-terminal (Slit2N) or C-terminal (Slit2C) domains, we performed co-IP experiments using HEK293 cells transfected with APP together with either FL Slit2 (Slit2) or its fragments Slit2N or Slit2C. Both the Slit2 and Slit2N coimmunoprecipitated with APP, but not the Slit2C fragment (Fig. 5*F*). Thus, APP interacts specifically with the N-terminal part of Slit similar to Robo-Slit binding (Nguyen Ba-Charvet et al., 2001). Finally, using P0 mouse brain lysates, we showed that APP could be coimmunoprecipitated by an anti-Slit2 antibody (Fig. 5*G*), demonstrating endogenous APP-Slit interaction *in vivo*.

Since APP and Robo both bind to Slit2N fragment, we asked whether APP and Robo could form a receptor complex. Cotransfection of FL human APP with HA-tagged Robo construct followed by IP with the APPc antibody indeed showed APP-Robo interaction (Fig. 5*H*). Reciprocal IP using an anti-HA antibody to immunoprecipitate Robo also pulled-down FL APP, but not APP mutant deleting the C-terminal domain (APPΔC; Fig. 5*I*). Further, coexpression of HA-Robo with APP C-terminal construct (APP-C99) and IP with the anti-HA revealed that APP-C99 coimmunoprecipitated with HA-Robo (Fig. 5*J*). The biochemical experiments using various APP mutants combined document that APP interacts with Slit through the E1 domain and Robo through its intracellular C-terminal domain (summarized in Figure 5*K*).

**Figure 6.**
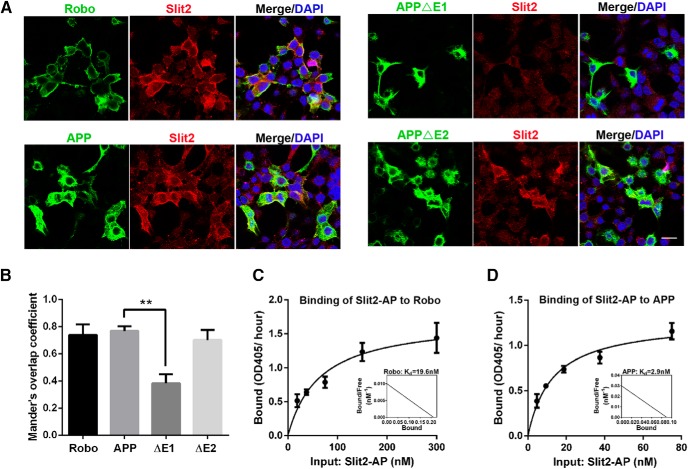
APP receptor and Slit2 ligand interaction by cell surface binding assay. ***A***, CMs from Slit2-Myc-expressing cells was applied to HEK293 cells transfected with either Robo, APP, APPΔE1, or APPΔE2 and incubated for 90 min. Robo- or APP-expressing cells were visualized by HA or 4G8 antibodies, respectively. Cell surface binding of Slit2 was detected with the anti-Myc antibody. Scale bar: 10 μm. ***B***, Quantification of the average proportion of Slit2 signals colocalized with Robo, FL APP, APPΔE1, or APPΔE2 using the plugin ‘Coloc 2’ of the Fiji software (ImageJ). **p < 0.01. ***C***, ***D***, Binding curves and Scatchard analysis of AP-tagged Slit2 binding to Robo (***C***)- or APP (***D***)-transfected cells. Binding experiments in lysates of HEK293 cells transfected with Robo or APP. The cells were incubated with increasing concentrations of AP-tagged Slit2. Binding affinity was calculated by measuring AP activity and described in Materials and Methods.

To corroborate the Slit-APP interaction detected by co-IP and GST pull-down, we performed fluorescent immunocytochemistry to examine whether secreted Slit protein could bind to the surface of cells in an APP-dependent manner similar to Robo (Fig. 6*A*). We transfected Robo or APP to HEK293 cells, incubated with supernatants of Slit2-Myc-expressing cells, and performed double immunofluorescence staining to visualize cells expressing Robo or APP and cells positive for Slit2 at the surface (Fig. 6*A*). Our results showed that, similar to Robo, Slit2 signal (red) can only be detected in APP-expressing (green), but not APP-negative (DAPI only) cells (Fig. 6*A*). Moreover, using constructs deleting either the extracellular E1 (APPΔE1) or E2 domains (APPΔE2) of APP, we found that APPΔE2, but not APPΔE1, showed APP and Slit2-Myc double-positive staining (Fig. 6*A*; and quantified in Figure 6*B*). The lack of Slit2 binding to the APPΔE1-expressing cells was not due to the reduced surface expression of APPΔE1 as the levels of cell surface protein expression of both APPΔE1 and APPΔE2 constructs have been characterized previously (Wang et al., 2009). These results are consistent with the biochemical interaction and reinforcing the notion that Slit2 binding is mediated through the APP E1 domain.

We next performed cell surface binding assays with alkaline phosphatase (AP)-tagged Slit2 and measured the affinity of APP and Slit2 binding. Using Scatchard plot, we calculated the *K*_d_ for Robo2 and Slit2 binding as 19.6 nM (Fig. 6*C*). Interestingly, APP binds to Slit2 at much higher affinity with a *K*_d_ of 2.7 nM (Fig. 6*D*).

### Slit binding triggers APP ectodomain shedding and intracellular complex remodeling

**Figure 7.**
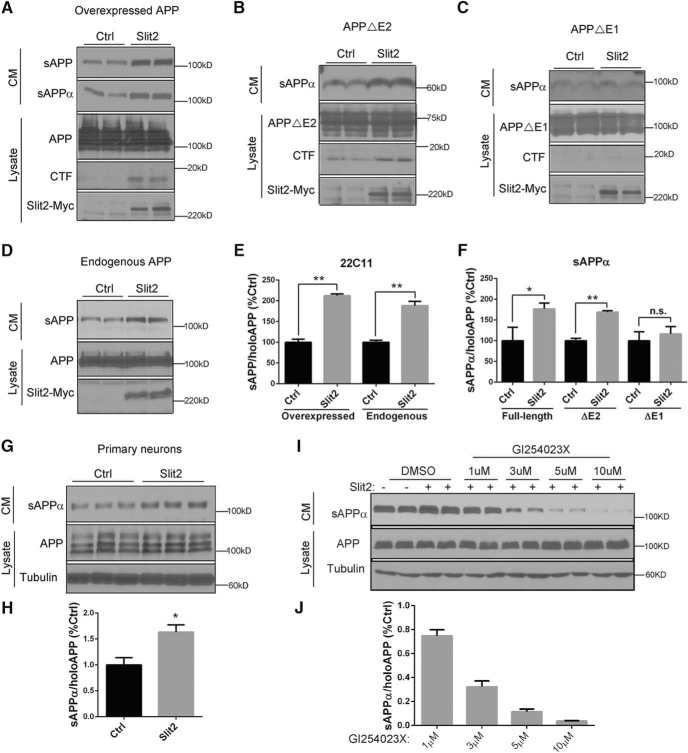
Slit binding triggers ectodomain shedding of APP. ***A***, Western blot analysis of CMs or cell lysates (Lysate) of HEK293 cells cotransfected with FL APP and empty vector (Ctrl) or Slit2. sAPP and sAPPα levels in the CM were measured by 22C11 and a sAPPα-specific antibody. FL APP and APP CTF were detected using the APPc antibody and Slit2 by the anti-Myc antibody. ***B***, ***C***, The same experiment as ***A*** except that APPΔE2 (***B***) or APPΔE1 (***C***) was used instead of FL APP. ***D***, Western blot analysis of endogenous APP in CMs or cell lysates (Lysate) of HEK293 cells transfected with empty vector (Ctrl) or Slit2. ***E***, Quantification of ***A***, ***D*** showing that Slit2 expression leads to increased sAPP from both overexpressed and endogenous APP. ***F***, Quantification of ***A–C*** showing Slit2-mediated increase of sAPPα by expressing FL APP and APPΔE2 but not APPΔE1. ***G***, Increased secretion of neuronal sAPPα from cocultures of primary cortical neurons with HEK293 cells expressing empty vector (Ctrl) or Slit2. Western blotting of CMs was blotted with a rodent-specific sAPPα antibody. ***H***, Quantification of ***G***. Calculated by normalizing sAPPα levels in CM to the APP levels of corresponding cell lysates. ***I***, ADAM10 inhibitor (GI254023X) suppresses Slit2-mediated APP ectodomain shedding. HEK293 cells were transfected with Slit2 or empty vector (−) and treated with DMSO or different concentrations of GI254023X. Production of secreted APP in CMs and corresponding APP expression in the cells were measured by Western blotting. ***J***, Quantification of I. *p < 0.05; **p < 0.01; n.s., nonsignificant (*t* test).

Many axon guidance receptors, including DCC and Robo, are proteolytically cleaved by Kuzbanian/ADAM10 family proteases at their juxta-membrane region leading to ectodomain shedding (Galko and Tessier-Lavigne, 2000; Coleman et al., 2010). ADAM10 is known to act as the α-site APP cleaving enzyme (Kuhn et al., 2010), which generates extracellular APPs (sAPPα) and a membrane-tethered CTF. We reasoned that this event may be mediated by Slit binding. To test Slit-mediated APP ectodomain shedding, we cotransfected HEK293 cells with APP and Slit2 or empty vector (Ctrl) and measured the production of secreted APP in CMs using the N-terminal antibody 22C11 that detect total soluble (sAPP) or an α-site cleavage-specific antibody for α-site cleaved (sAPPα) APP derivatives. Indeed, in cells cotransfected with APP and Slit2, there was a significant increase in total sAPP and sAPPα (Fig. 7*A* and quantified in Figure 7*E*,*F*), with a concomitant increase of APP-CTF in the corresponding cell lysates (Fig. 7*A*), demonstrating that Slit2 stimulates α-secretase cleavage of overexpressed APP. Consistent with the notion that APP E1 domain mediates Slit binding, cotransfection of Slit2 with APPΔE2 led to similar effect as the FL APP (Fig. 7*B* and quantified in Figure 7*F*), whereas no appreciable increases in sAPPα or CTF were detected on expressing APPΔE1 (Fig. 7*C* and quantified in Figure 7*F*).

To test whether the results obtained from the APP overexpression system also apply to endogenous APP, we transfected control or Slit2 vectors to HEK293 cells followed by Western blotting of CM for sAPP produced from endogenous APP (Fig. 7*D*). We found similar increases of sAPP without APP overexpression (Fig. 7*E*). Next, we assayed the effect of Slit2 on endogenous APP processing in primary neurons by coculturing the control or Slit2-overexpressing HEK293 cells with cultured primary neurons. Endogenous mouse sAPPα produced from the neuronal cultures (but not from the human HEK293 cells) was detected using a rodent-specific sAPPα antibody and normalized to total APP in the lysate of primary neurons (Fig. 7*G*). An increase in neuronal sAPPα was detected on coculturing with Slit2-transfected HEK293 cells (Fig. 7*G* and quantified in Figure 7*H*). Lastly, we tested whether Slit-mediated APP ectodomain shedding can be suppressed by α-secretase inhibition. HEK293 cells were transfected with Slit2 (+) or empty vector (−) (Fig. 7*I*). Twenty-four hours after transfection, fresh media with different concentrations of ADAM10 inhibitor (GI254023X) or DMSO controls were applied to the cells and the productions of secreted APP in CM from endogenous APP in the cells were measured. We observed reduced APP secretion in a dose-dependent manner in Slit2-transfected HEK293 after GI254023X treatment (Fig. 7*I* and quantified in Fig. 7*J*), confirming the essential role of ADAM10 in this process. The data combined provide strong support that Slit binding to the APP E1 domain triggers APP ectodomain shedding and sAPPα secretion through ADAM10 family proteases.

**Figure 8.**
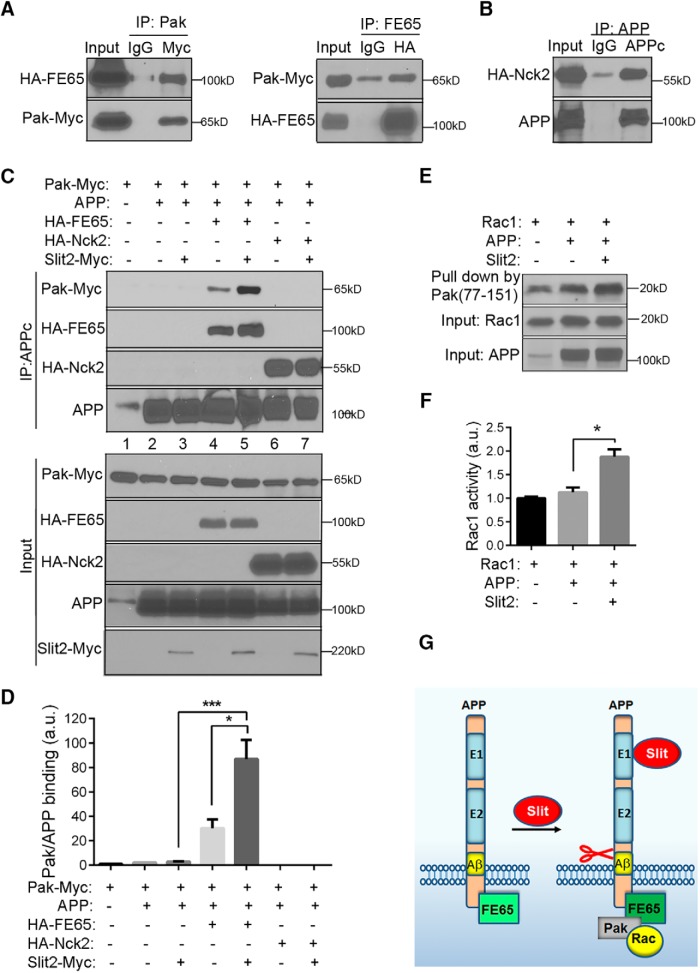
Slit2 promotes intracellular APP, FE65, and Pak complex and Rac activation. ***A***, Cotransfection of HA-FE65 and Pak-Myc in HEK293 cells followed by IP-Western blotting showed co-IP of FE65 and Pak-Myc. ***B***, Lysates of HEK-293 cells cotransfected with HA-Nck2 and APP were subjected to IP using the APPc antibody and Western blotting for HA-Nck2 and APP. ***C***, Cotransfection of HEK293 cells with APP and Pak1, with or without HA-FE65 or HA-Nck2, and IP with APPc showed no interaction between APP and Pak (lanes 2 and 3). Cotransfection with FE65 showed that both FE65 and Pak were pulled-down with APPc (lane 4), which can be further enhanced by treating the cells with Slit2 (compare lane 5 with 4). In contrast, cotransfection with HA-Nck2 did not promote the binding between APP and Pak (lanes 6 and 7). ***D***, Quantification of APP and Pak binding as shown in C. a.u., artificial units. ***E***, Slit2 promotes Rac1 activity mediated by APP. HEK293 cells were transiently transfected with APP and Rac1 expression vectors and treated with Slit2 for 10 min. The active form of Rac1 was pulled-down using GST-Pak (aa77-151), the active Rac-interacting domain of Pak. The precipitated active Rac1 was detected using the anti-Rac1 antibody. Expressions of Rac1 and APP were confirmed by Western blotting using the anti-Rac1 antibody and 6E10 (input). ***F***, Quantification of Rac1 activity normalized to Rac1 input was performed using ImageJ. *p < 0.05; ***p < 0.001. ***G***, A model for Slit2 triggered APP ectodomain shedding and intracellular signaling complex remodeling in axon guidance. Slit2 ligand binding to extracellular E1 domain promotes APP cleavage and release of APPs. The binding of Slit2 leads to the recruitment of FE65 and Pak to the cytoplasmic tail of APP and association of Pak with activated Rac1 to trigger downstream events essential for actin cytoskeleton dynamics.

Next, we sought to investigate the intracellular mechanisms downstream of APP in respond to Slit binding. On ligand-receptor interaction, activation of cytoplasmic signaling and cytoskeleton remodeling is critical for neurite outgrowth and axon guidance. The serine/threonine p21-activated kinase 1 (Pak1) is a key molecule in actin cytoskeleton assembly/disassembly, and its activation is under tight control through membrane recruitment of Nck family of adaptor proteins composed of Nck1 and Nck2 (Bokoch, 2003). A number of guidance receptors have been shown to associate with Nck, including Robo (Fan et al., 2003), DCC (Li et al., 2002), and the Eph receptors (Stein et al., 1998). Whether APP can recruit similar complexes is not known. Previous studies, however, have identified the adaptor protein FE65 as the major intracellular interacting partner linking APP to actin cytoskeleton remodeling (Borg et al., 1996; Bressler et al., 1996). To investigate which adaptor molecule, FE65 or Nck, serves as intracellular mediator downstream of APP-Slit2 interaction, we first tested whether FE65 can associate with Pak1 by performing co-IP in cells cotransfected with Myc-tagged Pak1 (Pak-Myc) and HA-tagged FE65 (HA-FE65). A strong association between FE65 and Pak1 was observed by IP for Pak-Myc or HA-FE65 and Western blotting for FE65 or Pak1, respectively (Fig. 8*A*), establishing FE65 as an adapter protein capable of linking Paks to membrane receptors. We then tested whether APP receptors can interact with Nck adaptors. Co-IP assays with cotransfected FL APP and HA-tagged Nck2 (Nck2-HA) revealed a positive interaction between APP and Nck2 (Fig. 8*B*). Next, to characterize APP and Pak1 interaction, FL APP and Myc tagged Pak1 (Pak1-Myc) were coexpressed, with or without HA epitope-tagged FE65 or Nck2. IP with the anti-APP antibody showed no detectable interaction between APP and Pak1 (Fig. 8*C*, lanes 2 and 3). However, a positive interaction was found when the cells were cotransfected with FE65 (Fig. 8*C*, lane 4). Thus, FE65 brings Pak1 to the APP, FE65, and Pak1 intracellular complex. Remarkably, treating the cells with Slit2 for 10 min before the IP resulted in much stronger interaction among APP, FE65, and Pak1 as shown by elevated Pak1 and FE65 protein levels in APP immunoprecipitants (Fig. 8*C*, compare lane 5 with 4; for quantification, see Figure 8*D*). These results demonstrate the intracellular APP-FE65-Pak1 complex formation that is modulated by extracellular ligand Slit2. In contrast, cotransfection with Nck2 with or without Slit2 treatment did not promote APP/Pak complex formation (Fig. 8*C*, lanes 6 and 7). These data demonstrate that FE65 modulates APP and Pak1 interaction independent of Nck and formation of the intracellular complex is facilitated by Slit binding to the APP EC.

Since Rac1, a widely expressed member of the Rho GTPase family, is a predominant signaling molecules of Pak that plays an essential role in actin cytoskeleton remodeling, we asked whether Slit2 binding triggers Rac1 activation by cotransfecting APP and Myc-tagged Rac1, pull-down with GST-Pak (aa77-151), which contains the active Rac-interacting domain of Pak, and Western blotting of the active form of Rac1 using the anti-Myc antibody. We observed a significantly increased activation of Rac1 after treatment with Slit2 (Fig. 8*E*, compare Slit2 – vs +; for quantification, see Figure 8*F*), suggesting that Slit2 binding to APP induces Rac1 activation.

In summary, our results support a model whereby Slit binding to the APP E1 domain triggers ectodomain shedding, leading to the release of APPs and recruitment of the APP, FE65, and Pak1 intracellular signaling complex and increased Rac1 small GTPase activity (Fig. 8*G*).

## Discussion

APP has long been speculated as a cell-surface receptor (Kang et al., 1987). However, although numerous candidate ligands for APP have been reported (Rice et al., 2013), none has been confirmed in a physiologically relevant context. Our genetic and functional analyses provide strong support that APP is a *bona fide* neuronal receptor for Slit that mediates axon guidance and circuit formation. First, we report a high frequency of malformation of the major forebrain axon track, CC, in CNS-specific *APP* and *APLP2* null mice. Second, we reveal that Slit2-induced axon repulsion in the 3D *ex vivo* olfactory explant culture is abolished in the absence of APP/APLP2, demonstrating a functional role of APP in mediating Slit effect. Last, inactivation of APL-1 in *C. elegans* results in pioneer axon guidance defects supporting a functional conservation, and genetic analysis places APL-1 in the SLT-1 (Slit)/SAX-3 (Robo) repulsive pathway independent of UNC-6 (Netrin)/UNC-40 (DCC)-mediated attraction.

The temporal and spatial expression patterns and biochemical characteristics provide additional support for the Slit/APP ligand receptor relationship. We reveal here that APP is highly expressed in developing axons and projection neurons and neuronal APP is known to be anterogradely transported (Koo et al., 1990) and targeted to the growth cone (Sabo et al., 2003), a motile structure that senses and executes directional cues present in the extracellular environment. Biochemically, most guidance receptors such as Robo and DCC function as multimeric complexes. APP also forms homophilic/heterophilic interactions, which have been shown to promote cell–cell adhesion and neurite outgrowth (Small et al., 1994; Soba et al., 2005). Further, dimerization of APP is mediated through the E1 domain and is induced on heparan sulfate binding (Dahms et al., 2010; Hoefgen et al., 2014). In this regard, it is interesting to note that Slit homodimer binds heparan sulfate and enhances Slit-Robo interaction and Slit repulsive activity (Hu, 2001). Thus, heparan sulfate may also participate in Slit-APP interaction and function. This common modality raises the possibility that APP could function alone to mediate Slit responses or it may be a component of a multimetic receptor complex including other receptors such as Robo. Indeed, many guidance receptors, such as Robo/DCC (Stein and Tessier-Lavigne, 2001) and DCC/UNC5 (Hong et al., 1999), form receptor complexes through direct interactions with each other to cooperatively respond to extracellular guidance cues. Our results that APP and Robo could form a receptor complex through the APP C-terminal domain further support this notion. In this regard, APP and Robo may either crosstalk through common C-terminal signaling complexes or engage independent intracellular signaling mediated by different adaptor proteins.

Although both APP and Robo bind to Slits, they may not directly compete for ligand binding as we found that APP binds to Slit2 at significantly lower concentrations than Robo (Fig. 6*C*,*D*), indicating that APP and Robo may respond to different Slit gradient. Our data suggest a model that growth cones with high expression of APP receptors sense low concentrations of Slits, whereas growth cones with high expression of Robo receptors sense high concentrations of Slits. Further studies are required to define the precise biochemical nature and cross talk among the different receptors.

Previous work have shown the resemblance of the CC defects in Robo1/Robo2 and Slit1/Slit2 double mutants, suggesting that Robo mediate the function of Slit1 and Slit2 in the formation of these connections. However, additional receptors have been implicated to account for Robo-independent signaling of Slits (Kidd et al., 1999; Lopez-Bendito et al., 2007; Jaworski et al., 2010). Our analysis raises the possibility that APP may mediate the Robo-independent function of Slits. Since Slits are involved in guidance of diverse axonal tracks including cortical axonal tracts, commissural axons in the spinal cord, retinal axons and OB axons, it is possible that APP/APLP2 dKO mice display other guidance deficits besides CC phenotype. A complete understanding the phenotypes and biochemical and genetic interactions require further investigation.

Our studies reveal that Slit binding triggers APP processing and ectodomain shedding. This event is likely mediated by ADAM10 proteases as ADAM10-specific inhibitor can block Slit2 triggered APP ectodomain shedding. Similar proteolytic cleavages have been observed for other guidance receptors including Robo, DCC, EphB2, and EphA4 (Bai and Pfaff, 2011). Interestingly, these receptors also undergo γ-secretase processing following ectodomain shedding. These sequential cleavages could thus act as a common mechanism to control guidance receptor activity through ligand-dependent activation followed by removal of membrane-associated fragments (Lichtenthaler et al., 2011).

We present evidence that the scaffolding protein FE65 is a positive regulator of the Slit-APP signaling downstream of Slit guidance cues. FE65 interacts with APP C-terminal sequences through its phosphotyrosine interacting domain (Fiore et al., 1995; Bressler et al., 1996), and has been postulated to facilitate the assembly of intracellular protein complex and modulation of actin dynamics. It has been reported that APP and FE65 colocalize to the dynamic adhesion sites within the growth cone lamellipodia to synergistically regulate growth cone motility (Sabo et al., 2003). Indeed, mice with deletion of FE65 and its homolog FE65L1 display CC defects that phenocopy the APP/APLP2 mutants (Guenette et al., 2006). Whether it is mediated in a Slit-dependent manner is an interesting question awaits further investigation. Altogether, these observations are consistent with the hypothesis that FE65 is an essential component of the Slit-APP pathway.

Pak is an effector of the Rac/GTPases that play essential roles in the regulation of cytoskeleton dynamics. A number of guidance receptors have been shown to associate with Pak, including Robo (Fan et al., 2003). Previous studies have established that Pak interacts with adaptor protein Nck, and binding of Nck to Pak1 is important for regulation of Pak1 subcellular localization and catalytic activity (Bokoch et al., 1996; Galisteo et al., 1996). We present evidence here that FE65 is a novel adaptor protein capable of recruiting Pak1 to the APP complex and this process is Slit dependent. It will be interesting to ascertain whether APP cleavage is required or promote APP-Fe65-Pak complex formation. Pak plays an important role in cortical synaptic morphology and memory consolidation (Hayashi et al., 2004) and may contribute to synaptic and cognitive deficits AD (Zhao et al., 2006). Our work raises the possibility that the APP/FE65/Pak signaling complex may be involved in these processes.

In adult brain under normal conditions, Slit-dependent axon guidance may not play prominent roles as neuronal axon pathfinding and circuit formation cease after development. However, under neuropathological conditions, such as traumatic brain injury (TBI) and AD, some of the developmental guidance processes may be re-activated. Indeed, it is known that injury to the adult CNS results in widespread changes in gene expression of guidance cues and their receptors (Curinga and Smith, 2008). In particular, Slit families and Robo receptors are expressed following lesions of the brain and spinal card (Wehrle et al., 2005). We and others have observed marked upregulation of axonal APP following TBI (Otsuka et al., 1991; Pierce et al., 1996; Guo et al., 2012). We also observed profound APP immunoreactivity in dystrophic neurites in the vicinity of amyloid plaques (Guo et al., 2012), supporting a potential role of axonal APP in mediating axon targeting and re-establishment of neuronal connectivity following neuronal insults. The functional role of APP axonal expression and whether Slit is involved in these processes need further investigation.

In summary, using a combination of genetic analysis in mice and *C. elegans*, an *ex vivo* axon guidance assay, and *in vitro* biochemical studies, we report here that the APP family of proteins function as a physiologic receptor for midline repellent Slit that mediates axon guidance and neuronal circuitry. Slit binding to the APP E1 domain triggers APP ectodomain shedding and promotes intracellular APP/FE65/Pak1 complex and Rac1 GTPase activation essential for cytoskeleton remodeling. In light of the central role of APP in AD pathogenesis, impairment of APP-mediated axon pathfinding may contribute to circuit dysfunction in AD.

## Synthesis

Reviewing Editor: Jorge J. Palop, Gladstone Institutes and UCSF Decisions are customarily a result of the Reviewing Editor and the peer reviewers coming together and discussing their recommendations until a consensus is reached. When revisions are invited, a fact-based synthesis statement explaining their decision and outlining what is needed to prepare a revision will be listed below. Note: If this manuscript was transferred from JNeurosci and a decision was made to accept the manuscript without peer review, a brief statement to this effect will instead be what is listed below.

N/A
